# Pearl's causality for integrating ecological datasets: A case study on *Myricaria germanica* in northern Italy

**DOI:** 10.1002/aps3.70056

**Published:** 2026-05-10

**Authors:** Kailin Weitkämper, Kilian Rückschloß, Tommaso Sitzia, Bruno Michielon, Silke Werth, Felix Weitkämper

**Affiliations:** ^1^ Faculty of Biology LMU University of Munich Menzinger Straße 67 München 80638 Germany; ^2^ Institute for Mathematics University of Tübingen Auf der Morgenstelle 10 Tübingen 72076 Germany; ^3^ Department of Land, Environment, Agriculture and Forestry Università degli Studi di Padova, Viale dell'Università 16 Legnaro 35020 PD Italy; ^4^ Research Center for Artificial Intelligence German University of Digital Science Marlene‐Dietrich‐Allee 14 Potsdam 14482 Germany

**Keywords:** applied ecology, conceptual framework, interdisciplinary science, *Myricaria germanica*, Pearl's causality

## Abstract

**Premise:**

Applied ecology can significantly influence policy decisions on environmental issues. Therefore, research in this field should be as transparent and reproducible as possible. Existing expertise from a broad range of disciplines should also be integrated into ecological research to allow researchers to maximize understanding of complex systems.

**Methods:**

We illustrate how Pearl's causality can contribute to applied ecology. We demonstrate the implications of causal diagrams for assessing the effects of anthropogenic and abiotic factors in ecological systems, using *Myricaria germanica* in Italian river systems as an example. In particular, we showcase the interplay between explicit causal modeling and classical statistical techniques.

**Results:**

In our example, we find that river channel width and riverbank protections are the most important factors affecting the survival of *M. germanica* juveniles in northern Italy. Other factors such as altitude and other human activity also impact *M. germanica* survival through channel width as a mediator.

**Discussion:**

We demonstrate that causal diagrams can be an effective new language for ecological research. The causal diagram highlights that integrating hydrological, historical, or anthropological data could strengthen understanding of *M. germanica* populations in river systems. This framework facilitates interdisciplinary research and realizes the full potential of ecological datasets.

Ecology is a field in which research output can have profound impacts on the environment. This potential is especially pertinent in light of the ongoing climate change and biodiversity crises. The ability to collect, analyze, and interpret ecological data regardless of habitat and ecosystem type is essential for informed decision‐making about conservation strategy and environmental policy (Bornmann et al., 2022). Given the scale of the potential consequences on environmental policy, interdisciplinary research is a huge advantage; there is an ongoing push towards interdisciplinarity in the scientific community (Ledford, 2015). Collaborations across disciplines have been shown to increase the quality of scientific thinking (Buettel et al., 2018); however, biodiversity science as a whole has not become more interdisciplinary, despite the importance of the field (Craven et al., 2019).

Ecological research comes with its own unique challenges, such as the unpredictability of working in nature. For example, ecological data are often very labor‐intensive to collect; the amount of data that can be collected is fundamentally limited because there are only so many suitable study plots that can be sampled. In addition, organisms of interest could be rare, protected, or inaccessible, resulting in sampling gaps.

Once the data are collected, the next challenge is to analyze the dataset. In a randomized controlled trial, as pioneered by Fisher (1935), researchers can control for variables and directly test the effects of a treatment. Meanwhile, researchers using observational data from fieldwork must use statistical methods to test for correlation rather than infer direct causation. This limitation becomes critical in the context of the broader reproducibility crisis, where scientific results cannot be repeated because of differences in analysis choice. In ecological research, a recent meta‐study found that over 200 researchers analyzing the same dataset came to wildly different conclusions (Gould et al., 2025). This is because decisions like which statistical test to use under which assumptions are often subjective. Using causal diagrams could facilitate more transparent analysis decisions, as put forward by Gould et al. (2025). In summary, the challenges facing ecology include difficulties in the collection of large datasets, limitations to experimental design, and the pitfalls of correlation‐based statistical methods. Here, we introduce a novel analysis tool that can alleviate some of the challenges in ecological research by making assumptions transparent and providing an interface for integrating independently obtained research outputs.

## *Myricaria germanica* ecology as a case study

**Figure 1:**
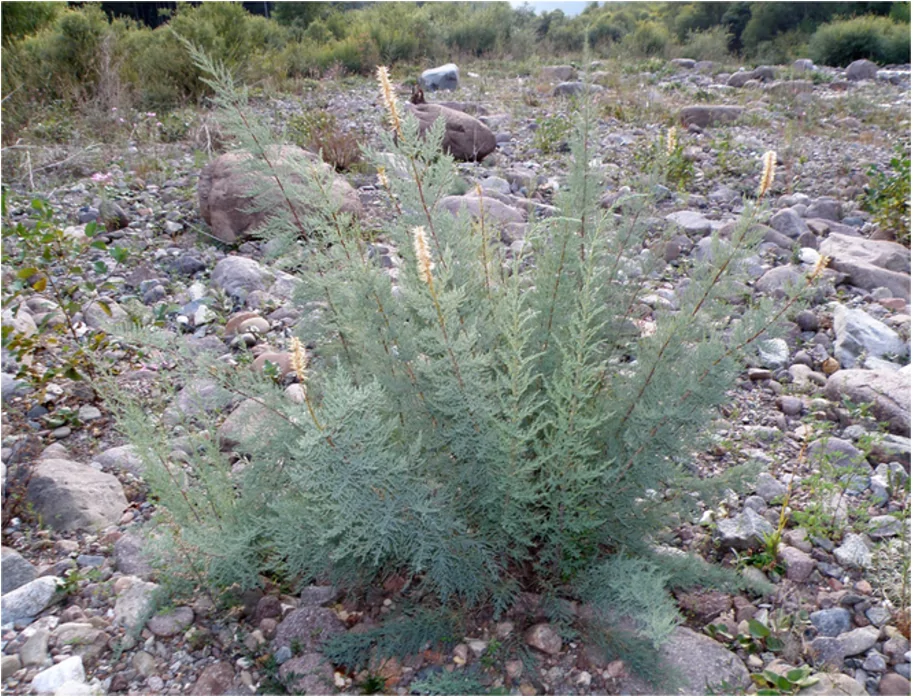
*Myricaria germanica* on a gravel bank in the study region (photo taken by Bruno Michielon).

Studying ecological processes is necessary for decisions about management, conservation, and policy. Given the diversity of habitats and ecosystems worldwide, focusing on only a few model organisms is insufficient. Furthermore, species or areas that are threatened often have the least pre‐existing resources that could be built upon, and are also the most in need of action. *Myricaria germanica* (L.) Desv. (Figure 1) is a prime example of a non‐model organism with ecological importance and an endangered conservation status. The genus *Myricaria* Desv. has a broad Eurasian distribution, with its center of origin in the Qinghai–Tibetan Plateau region, from which *M. germanica* spread westward (Sun and Werth, 2024). Today, *M. germanica* is the only representative of the genus in Europe (Webb, 1968).

This species can be found in alpine and riparian regions throughout Europe, such the Central European Alps, the Pyrenees, the Balkans, and Scandinavia (GBIF, 2025). The NATURA 2000 habitat framework (European Environment Agency, 2013), created by the European Union's Habitats Directive, provides a useful definition of *M. germanica* habitat in the European Union. In this framework, *M. germanica* is found in the habitat type “Alpine rivers and their ligneous vegetation with *Myricaria germanica*” (habitat code 3230), with a geographic distribution including Austria, Finland, France, Germany, Italy, Spain, and Sweden. This habitat type corresponds to habitat C3.55 (“Sparsely vegetated river gravel banks”) in the Interpretation manual of the habitats listed in Resolution No. 4 (1996) (Evans and Roekaerts, 2019). For the period 2013 to 2018 (the most recently reported period), the Habitats Directive reports the conservation status of habitat type 3230 as “unfavourable to bad” in Italy, the focus region of this study (European Environment Agency, 2025). In the Italian Red List of plant species, *M. germanica* is listed as endangered (Rossi et al., 2013). Its habitat type is described as “alpine and pre‐alpine riparian forest and shrubland” (C26) in the Red List of Italian Ecosystems (Blasi et al., 2023), where it is also listed as endangered.

The unique life cycle of *M. germanica* (Sitzia et al., 2021) on gravelly banks of free‐flowing rivers makes it a pioneer species of this habitat. Seeds can quickly germinate in suitable conditions, the plants have flexible branches that are able to withstand floodwater, and they can rapidly regenerate from damage and burial (Kudrnovsky, 2013). In addition, the roots of *M. germanica* help reduce soil erosion, enabling other species to establish in the area. Survival of *M. germanica* is only possible in environments where the gravel turnover is high, so they are also considered to be indicator species for riparian habitats (Harzer et al., 2018). The presence of *M. germanica* is highly correlated with many threatened or rare insect species, such as *Tetrix tuerki*, *Chorthippus pullus*, *Merulempista cingillella*, and *Bryodemella tuberculatum*, as well as bird species including the little ringed plover (*Charadrius dubius*) and the common sandpiper (*Actitits hypoleucos*) (Kudrnovsky and Stöhr, 2013). For this reason, *M. germanica* is also considered a keystone species. The importance of *M. germanica* as a pioneer, indicator, and keystone species means that this species and its associated habitat types are protected (European Environment Agency, 2013; Rossi et al., 2013; Blasi et al., 2023). Unfortunately, these habitats are threatened by anthropogenic activities, such as river straightening and dam building (Werth and Scheidegger, 2014; Werth et al., 2014; Sitzia et al., 2016). In China, a related species, *Myricaria laxiflora* (Franch.) P.Y. Zhang & Y.J. Zhang, was thought to be extinct due to the construction of the Three Gorges Dam (Liu et al., 2006). Efforts to inform conservation policy should therefore understand the mechanisms by which human activities reduce *M. germanica* populations.

To elucidate the effects of different variables on *M. germanica* populations, data have been collected across 133 sites in several river basins in northern Italy (Sitzia et al., 2016; Michielon and Sitzia, 2019). In this region, rivers have been subject to long‐term alteration of their geomorphological patterns due to channelization, flow regulation, and sediment mining, resulting in reduced river corridor width, sediment deficits, and simplification of channel forms (Comiti, 2011). The collected data include geographic coordinates, river features, altitude, and numbers of juvenile plants. These data points, recorded annually from 2009 to 2020, provide valuable information on the presence and absence of *M. germanica* over a wide geographic area and over a period of a decade, even though some records are incomplete. In the context of understanding *M. germanica* ecology and conservation in northern Italy, we used this dataset as the basis for our case study applying Pearl's causality to ecological questions. Although this dataset was very labor‐intensive to collect, it is still small compared to the optimal dataset size for most machine learning approaches, and distinguishing different hypotheses with statistical significance remains a challenge. This type of “small data” can be very important to ecology researchers, as data on rare species like *M. germanica* are difficult to collect and, by definition, limited (Todman et al., 2023).

As is typical of time‐series ecological datasets, population data are available for multiple years at each site, whereas site‐specific variables are recorded only once. On the other hand, even sites with incomplete or missing population data can still provide valuable information about relationships among the site‐level factors. Making full use of all available data points, even those that only provide partial information, is essential to getting the most value out of such a dataset.

## Causality in ecology

The method of causal diagrams which we advocate for here is inspired by path analysis (Wright, 1921), which decomposes (traditionally linear) effects along the paths of a diagram. Path analysis is widely used in the biological sciences, including in ecology (Shipley, 1997). While Pearl's notion of causality and traditional path analysis are clearly related, they differ in that path analysis (and its generalization structural equation modeling) treats structural equations as the key formal concept and derives the associated diagram from the equations. Originally, the diagrams were merely seen as an intuitive shorthand; more recently, Pearl (2012) and Shipley (2016) adapted methods from Pearl (2000) to infer specific dependencies from the structure of the diagrams.

In contrast, our approach treats causal diagrams as independent entities that carry meaning beyond the specific equations employed. This distinction is particularly crucial where categorical variables and/or non‐linear dependencies are involved. Binary variables, for instance, are usually estimated by a form of generalized linear model, such as logistic regression. The natural outcome of a logistic regression analysis is a formula such as

(1) $$\text{Prob}(Y=1)≔\text{some}\;\text{formula}\;\text{involving}\;X_{1}\;\text{and}\;X_{2}$$

specifying the probability that the target takes a certain value, rather than a formula of the form

(2) $$Y≔\text{some}\;\text{formula}\;\text{involving}\;X_{1}\;\text{and}\;X_{2}$$

as required for a structural equation. Adopting causal diagrams as a tool in their own right allows us to adopt Bayesian network semantics, where the values taken by feature variables determine the distribution of the target variable but are not required to determine the actual value taken by the target variable.

Another notion of causality encountered in ecology is that of Granger (1969); this is based on the common sense that causes precede their effects and allows one to test whether, for example, temperature should be considered a cause of plant growth. Essentially, temperature is considered a cause of plant growth if knowledge of past temperature values improves predictions of future plant growth. Applications of Granger causality to ecological research include modeling species‐to‐species interactions (Barraquand et al., 2021); modeling pest populations, temperature, and humidity in an agricultural landscape (Damos, 2016); and interpreting the dynamics of the photosynthesis–soil respiration system (Detto et al., 2012). While methods based on Granger causality are useful for testing causal relationships using time series, it does not make use of explicit causal diagrams, which is the key tool in our study.

## On Pearl's notion of causality and causal diagrams

**Figure 2:**
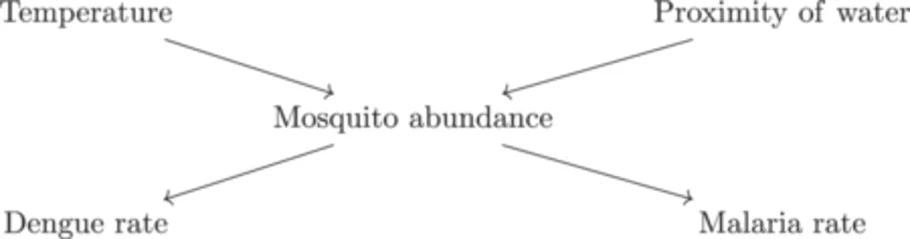
Fictitous example causal diagram showing the relationships among variables affecting the rates of the infectious diseases malaria and dengue fever.

Pearl (2000) provides a different account of causality that allows for causal reasoning based on assumptions about the directions of influences between variables. Recently, several authors proposed the use of Pearl's causality to answer ecological questions (Arif and MacNeil, 2023; Correia et al., 2025, 2026). The key innovation of Pearl's (2000) theory is the use of *causal diagrams*, which capture dependencies and causal ordering. Pearl's approach can be considered a generalization of Sewall Wright's (1921) method of path analysis. A sample causal diagram, a very simple example from the field of epidemiology, is shown in Figure 2; causal diagrams like this example capture two different kinds of information. First, they capture (in)dependencies (correlations). For instance, if Figure 2 correctly captures the causal mechanisms at play, then we expect the temperature and the existence of bodies of water to be independent. On the other hand, we expect dengue and malaria rates to be correlated, because they share a common cause. In areas of high abundance of mosquitoes, both diseases are expected to be frequent, and in areas of low abundance both are expected to be low. However, once we control for mosquito abundance, the situation is reversed. Temperature and water proximity are now generally correlated; if we look only at situations of low mosquito abundance, then it may seem as if bodies of water only lie in cold areas, because areas that are both moist and warm have been excluded (they would have had too many mosquitoes). It is clear that, given this diagram, temperature and malaria rate will generally be correlated, as temperature affects mosquito abundance, which in turn affects the incidence of malaria. In this constellation, mosquito abundance is a *mediator* for this effect. In fact, the diagram also implies that the only way temperature affects malaria rates is by affecting mosquito abundance, and therefore that malaria rate and temperature are independent when conditioning on the abundance of mosquitoes. The complete set of rules for when variables in a causal diagram are rendered independent is known as *d*‐separation and is explained in full detail by Pearl (2000). Shipley (2016), Pearl and Mackenzie (2018), and Correia et al. (2026) provide introductions aimed at non‐specialists.

A second type of information captured by causal diagrams is information about what happens when some variables are changed from outside the system. In the causal diagram of Figure 2, the abundance of mosquitoes and the incidence rate of malaria are clearly correlated. When intervening in this system by killing mosquitoes, this impacts the rate of dengue (which should drop accordingly). However, if we decrease the rate of dengue directly by administering a vaccination, for example, this has no impact on the mosquito population. In this sense, causal diagrams express assumptions not only about the variables we can observe, but also about how external changes propagate.

So, while observational data can be used to refute a proposed causal diagram (by refuting its independence statements), it can generally not be used to generate one. Orienting the arrows of a causal diagram still requires human intuition or domain knowledge. Often this is very easy: certainly, the abundance of mosquitoes does not affect the average temperature, and it is the mosquitoes that lead to an increase in malaria rather than the illness increasing the mosquito population. In other cases, deciding causal direction can be less straightforward. An important consideration about causal diagrams is that they do not imply that a variable depends *only* on those other variables with arrows pointing into it. For instance, there are many factors affecting mosquito abundance apart from temperature, like the abundance of predators or ecological competitors, and this alone does not invalidate the diagram. However, using the diagram to express effects does assume that any additional factor affecting mosquito abundance is not itself a cause of any of the remaining entries in the diagram. This is usually phrased as the absence of *hidden confounders*. For instance, if poor sanitation leads to an increase in mosquito numbers *and* to an increase in dengue rates, our observations may overestimate the effect of mosquitoes on dengue because they do not control for that confounding variable.

Causal diagrams allow us to evaluate direct, indirect, and total effects of one variable on another. The total effect of a feature on a target is the total change to the target when the feature is manipulated. In Figure 2, the total effect of the proximity of water on malaria rate can be decomposed into the effect of water location on mosquito abundance and the effect of mosquito numbers on the incidence rate of malaria. However, as explained above, the diagram also implies that the entire effect of water proximity on the incidences of the diseases is mediated by mosquito abundance; in other words, there is no *direct effect* of water sources on malaria. The notion of a direct effect only makes sense relative to a given set of variables. For instance, Figure 2 implies that proximity of water has a direct effect on mosquito abundance, but if one were to add an additional variable representing abundance of larvae, this may well mediate the entire effect of water proximity on adult mosquitoes, which would then be seen as indirect.

A particular feature of ecological research is that the factors included in a study are often the purview of other disciplines. For instance, the fate of an ecosystem will generally depend on both natural and anthropogenic aspects of their environment. Those features in turn are more commonly studied by other scientists such as geographers, agronomists, or social scientists. Because applied ecology and conservation are complex fields with many interlinking factors, interdisciplinary research is key to protecting biodiversity and ecosystems. Despite the best efforts of the scientific community, there are various practical and theoretical barriers to interdisciplinary research in conservation biology and the environmental sciences (Boulton et al., 2005; Campbell, 2005; MacLeod and Nagatsu, 2018). A major benefit of using causal diagrams as a framework is that it allows scientists to partition a problem into its constituent parts. This creates an interface for scientists from different fields to combine their contributions.

The concrete realization of causal diagrams are *Bayesian networks*, which specify the probability distribution of a target variable as a function of the values of those feature variables whose arrows point into the target. Nodes without any arrows pointing into them are just prescribed a single probability distribution. In our study, we use well‐known regression models for this purpose, namely *linear regression* in the case of continuous target variables and *logistic regression* in the case of binary target variables. However, the theory of Bayesian networks is fully general and does not rely on linearity in any way.

**Figure 3:**
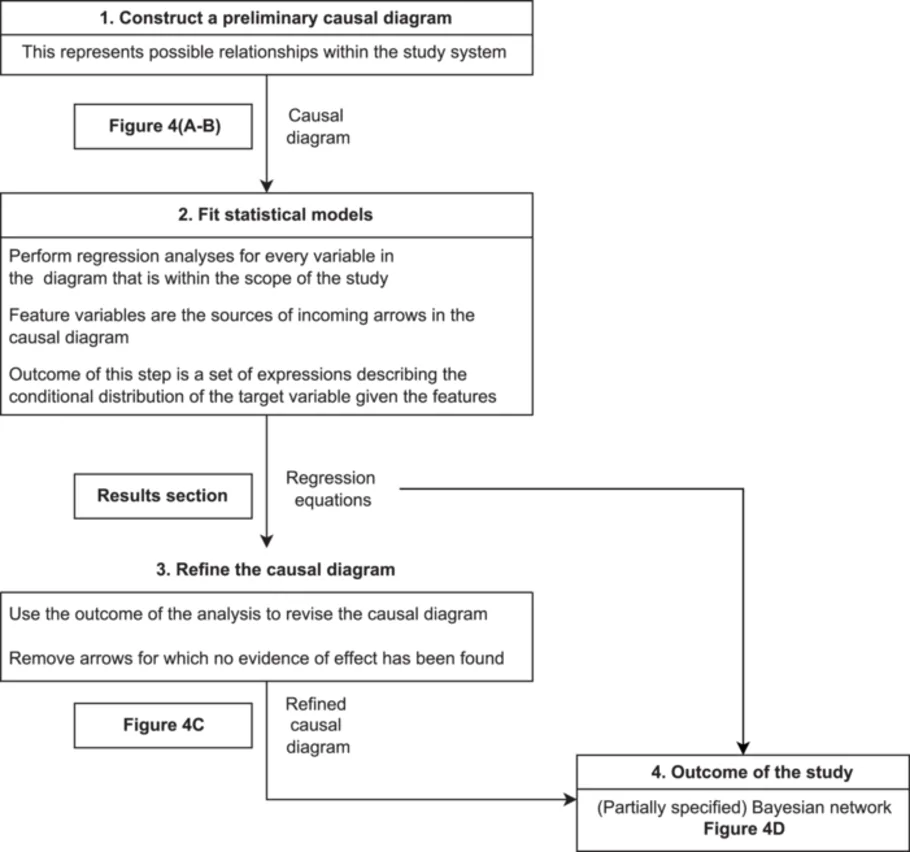
A roadmap of this case study detailing how causal diagrams are used to understand variables affecting *Myricaria germanica* survival in northern Italy.

A logistic regression model expresses a dependency between numerical feature variables *X*_1_,…,*X*_*n*_, taking values *x*_1_,…,*x*_*n*_ and a binary response variable *Y*, by modeling the response as a Bernoulli trial with probability Prob(*Y*) = sigmoid(*α*_0_ + *α*_1_ *x*_1_ + ··· + *α*_*n*_ *x*_*n*_) for some parameters *α*_0_,…,*α*_*n*_ that are estimated from data. A linear regression model treats a continuous response *Y* as normally distributed, with a mean *α*_0_ + *α*_1_ *x*_1_ + ··· + *α*_*n*_ *x*_*n*_ and a standard deviation *σ*, which is estimated from the residual sum of squared errors after fitting the parameters *α*_0_,…,*α*_*n*_. In both cases, binary feature variables such as the presence of a check dam or concrete riverbanks are accommodated using indicator variables, where for a variable *X* (which can be true or false) the indicator 1_*X*_ = 1 if *X* is true and 1_*X*_ = 0 if *X* is false. In this contribution, we demonstrate how causal diagrams, according to Pearl's notion of causality, can be used in applied ecology. We use the real‐world example of the disappearance of juvenile populations of *M. germanica* (a non‐model organism) in Italy to illustrate the potential of this conceptual framework. By investigating factors like altitude, river channel width, and other human activity, we show that complex interactions can be modeled using causal diagrams. We also discuss the potential of this concept for facilitating interdisciplinary research and describe how causal diagrams can be used to analyze ecological data. A brief roadmap of this process is summarized in Figure 3. Here, we describe each of these steps in the context of our *M. germanica* case study. Details on executing steps 1 and 2 can be found in the Methods section, while the results of our regression analyses are presented in the Results section. We present steps 3 and 4 in the Discussion section.

## METHODS

### Data sources and collection

The data for this case study was collected in northern Italy between 2009 and 2020 in the Adige, Piave, and Tagliamento river basins. A synthesis of the *M. germanica* population data for these river basins is reported in Table 1.

**Table 1:** Overview of the population data underlying this study (Sitzia et al., 2016; Michielon and Sitzia, 2019, 2025).

River basin	Province(s)	Sites with *Myricaria germanica*	Extinct sites	Total sites	Mean altitude (m a.s.l.)	Mean corridor width (m)	Total no. of plants	Mean plants/site
Adige	BZ, TN	47	33	80	1187	45	15,585	332
Piave	UD, BL	20	9	29	839	154	13,440	672
Tagliamento	UD	27	5	32	333	465	35,941	1123
Total/mean		94	52	146	892	264	64,966	653

*Note*: BZ = Bolzano; BL = Belluno; TN = Trento; UD = Udine.

Detailed information about the study sites and the definitions of the different features are reported by Sitzia et al. (2016) and Michielon and Sitzia (2019). Overall, the dataset used here covers 133 sites and partially annual data (not every site is recorded in every year) of *M. germanica* populations across the study period.

### Constructing a preliminary causal diagram

Here, we demonstrate for our case study how a causal diagram is constructed. The process leading to a causal diagram starts with variable selection. In this study, we have a number of different sites and years, and we have data on the existence of juvenile and adult *M. germanica* plants for most combinations of site and year. We also have data on the altitude; the placement of check dams, concrete riverbanks, and flood levees; the protection status of a site; and the width of the active river channel at each site.

#### Modeling the variables

We made two key choices on modeling the variables we investigated in our study. The first is conceptual: anthropogenic activity follows intricate patterns, where dependencies between different human actions are very hard to decipher. Hence, rather than positing causal relationships between anthropogenic factors, we view them as induced by a latent variable, which we call *human factors*. The second is more concrete: rather than treating the abundance of *M. germanica* as a continuous variable, we decided to treat the outcome as the event of *disappearance* from either natural dynamics or anthropogenic factors. This is meaningful because juveniles indicate recent germination and successful recruitment; their loss indicates that current conditions no longer support germination or establishment. Adult plants, which can persist for decades (up to 70 years) (Frisendahl, 1921; Sitzia et al., 2016), are less sensitive to short‐term disturbance, so remnant adult stands may remain even when recruitment has ceased. So, our target variable is the disappearance of juveniles in a given time‐step, that is, the presence of juveniles in Year *n* and their absence in Year *n* + 1. This recognizes the biologically very meaningful clustering of population numbers at zero (sites with no [juvenile] plants).

**Figure 4:**
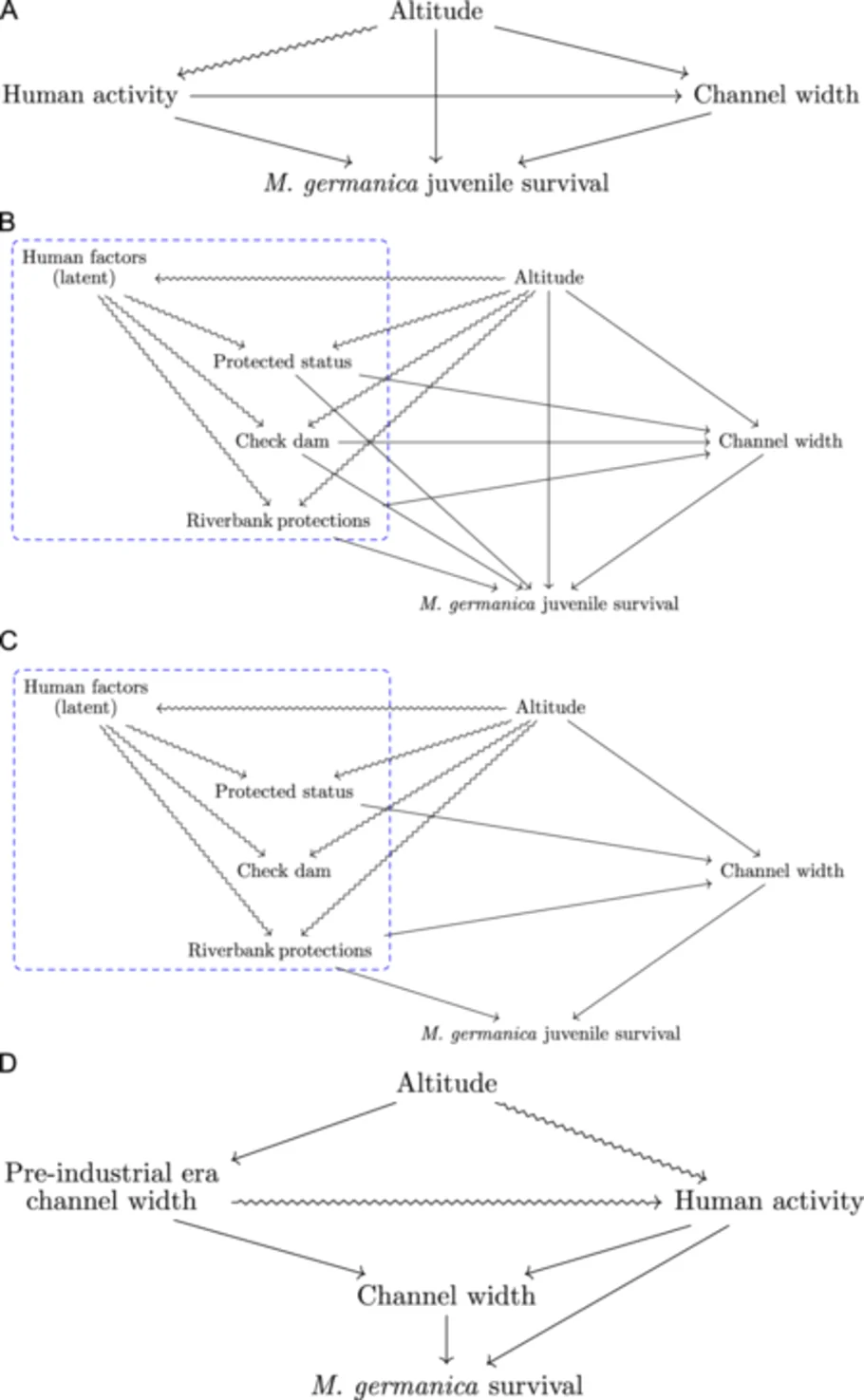
The development of a causal diagram to model various effects on *Myricaria germanica* juvenile survival in northern Italy. Effects estimated in this study are represented by straight lines; other effects are represented by wavy lines. (A) A conceptual model causal diagram of the variables represented in our dataset. (B) The causal diagram of (A) extended to include details of human factors, which are enclosed in a dashed box. (C) The causal diagram of (B) with straight lines only where effects have been detected. Human factors are enclosed in a dashed box. (D) A postulated causal diagram to guide future research.

Thus, the simplified diagram of Figure 4A contains four variables, namely altitude, channel width, human activity, and the juvenile survival of *M. germanica* populations. As explained above, plotting those variables into a self‐contained causal diagram assumes that all dependencies that we see come from actual relationships rather than from hidden confounders. This can be a strong modeling assumption, and the causal diagram may have to be refined later if such hidden confounders are suspected. Explicitly displaying the available human factors yields Figure 4B, with the posited latent variable as their common confounder.

It is important to recognize that the inclusion of certain variables in a diagram does not imply that those factors are the only ones of importance. For instance, *M. germanica* populations are constrained by the abundance of competitors such as willows of the genus *Salix* L. (Sitzia et al., 2021). However, this does not invalidate the diagram as *Salix* abundance is not itself a cause of human activity, altitude, or channel width—in other words, it is not a confounder of the variables in the model. This does not preclude the possibility that *Salix* is itself affected by the width of the active channel and that one of the reasons for the dependence of *M. germanica* survival on the active channel is through its effect on the *Salix* population. In that case, it would be a mediator of the effect of channel width on *M. germanica* rather than a confounder.

#### Understanding directionality of causes

Initially, all variables are connected by arrows, because there is no theoretical reason to assume that these variables are not causally related. Our analysis will demonstrate whether it is possible to detect those dependencies in our data. This still leaves open how to orient the arrows in our diagram. For some variables, this is clear from common sense: altitude is fixed regardless of any external factors, so it cannot have any incoming arrows. For other variables, we can make reasonable assumptions, but they are not necessarily entirely straightforward. We assume that channel width and human activity affect plant survival and not vice‐versa, because the channel width has been measured once at the beginning of the study. Responses of the active channel or of human activity to subsequent plant survival can therefore be ruled out. Had we used a quantitative response such as the raw number of *M. germanica* plants, this would have been less clear, because vegetation density does in general affect the active channel as well as the feasibility of engineering projects. Lastly, we assume for our analysis that human activity causes changes in the active channel and not vice‐versa. At first glance, this seems reasonable because there is ample evidence, particularly from our study region, that human activity has had a strong effect on river morphology in the preceding 200 years (Rinaldi et al., 2005; Surian et al., 2009; Ziliani and Surian, 2012; Huber and Huggenberger, 2015; Ziliani and Surian, 2016). However, it is equally well known that human settlement patterns closely follow rivers, which are crucial for transport and irrigation. Therefore, there could be complex inter‐relationships between river morphology and human behavior that are not expressed in Figure 4A.

#### Clarifying the scope of our analysis

Once we determine our proposed causal diagram, we can fit regression models to our data. Whether we fit models for all response variables or only for some of them will depend on our research question. Ecologists are interested in understanding direct effects of non‐anthropogenic abiotic factors on plant survival, in this case altitude, in order to understand the natural effects of the habitat type on the study organism. Conservationists are interested in the total effect of human activity on plant survival, because human activity may be shaped and regulated through policy. However, the aggregate effect of altitude (including through its impact on human activity by influencing patterns of settlement or pasture) is less immediately useful, because it is not possible to change the altitude at any given site. Accordingly, we estimate only those effects represented in Figure 4 by straight lines. In causal modeling for applied natural sciences, one is typically more interested in arrows going out from anthropogenic factors than in arrows coming in; the incoming arrows are usually considered the remit of the social sciences. Of course, estimating the effect of human activity on the width of the active channel, for example, is a question that plant scientists are not well equipped to answer. Thus, our causal diagram is also an opportunity to interface with hydrologists and geomorphologists who have expertise in analyzing those relationships.

#### Selecting model spaces

Having decided on the underlying causal diagram and selected which response variables to investigate, one can now use approaches from machine learning and statistics to fit regression models for those response variables. For *M. germanica* survival, which is a binary response variable, we fit a logistic regression model. The channel width is continuous, but with the added complexity that it varies widely but must never be negative. This suggests a log‐linear model as a first approach, where a linear model is fitted to the logarithm of the target.

### Statistical analyses

#### Estimating the disappearance of juvenile plants

Following the causal diagram, we investigated the probabilities of the disappearance of juvenile *M. germanica* during any year where this could reasonably be detected in our data (i.e., juveniles are known to be present in this year, and the presence of *M. germanica* in the following year is also known). To test possible effects of the river properties on *M. germanica* populations, we performed a logistic regression by applying the iteratively reweighted least squares algorithm (Nelder and Wedderburn, 1972; Bishop, 2007) with every subset of the following feature variables: (1) altitude – continuous; (2) width of the active channel – continuous; (3) protection status of the site – binary; (4) riverbank protection – ternary; and (5) the presence of check dams – binary. Because riverbank protection is an ordered variable with levels “none,” “non‐concrete,” and “concrete riverbanks,” we model them here and in the log‐linear regression below with one indicator variable for “some riverbank protection” (RBP) and one indicator variable for “concrete riverbank protection” (CRB). We performed feature selection using the Aikake information criterion (AIC) (Akaike, 1973, 1974), selecting the combination of feature variables that has the lowest AIC value. Additionally, we assessed the significance of every feature included in that model using analysis of deviance (Wilks’ likelihood‐ratio test; Wilks, 1938) by comparing the full model to a model including all but that one feature.

#### Estimating channel width

We then performed a log‐linear regression to investigate the impact of the remaining features on the channel width. More precisely, we used the normal equations to solve linear regression models using the following feature variables: (1) altitude – continuous; (2) protection status – binary; (3) riverbank protection – ternary; and (4) the presence of check dams – binary. We also included the terms resulting from multiplying any of the features with altitude, including a squared altitude term. In this analysis, we included all sites for which the corresponding data were available, including those without natural populations of *M. germanica*. As a target variable, we used the logarithm of the channel width. We performed variable selection and significance tests as above, using *F*‐tests to assess the significance.

#### Assessing the association between human factors

We used Pearson's *χ* ^2^ test and Cramér's V to assess the pairwise correlation between the presence of check dams, protected status, and the presence of riverbank protections. We viewed riverbank protections as a variable with three levels (i.e., none, plain, and concrete riverbanks).

## RESULTS

### Population survival

**Figure 5:**
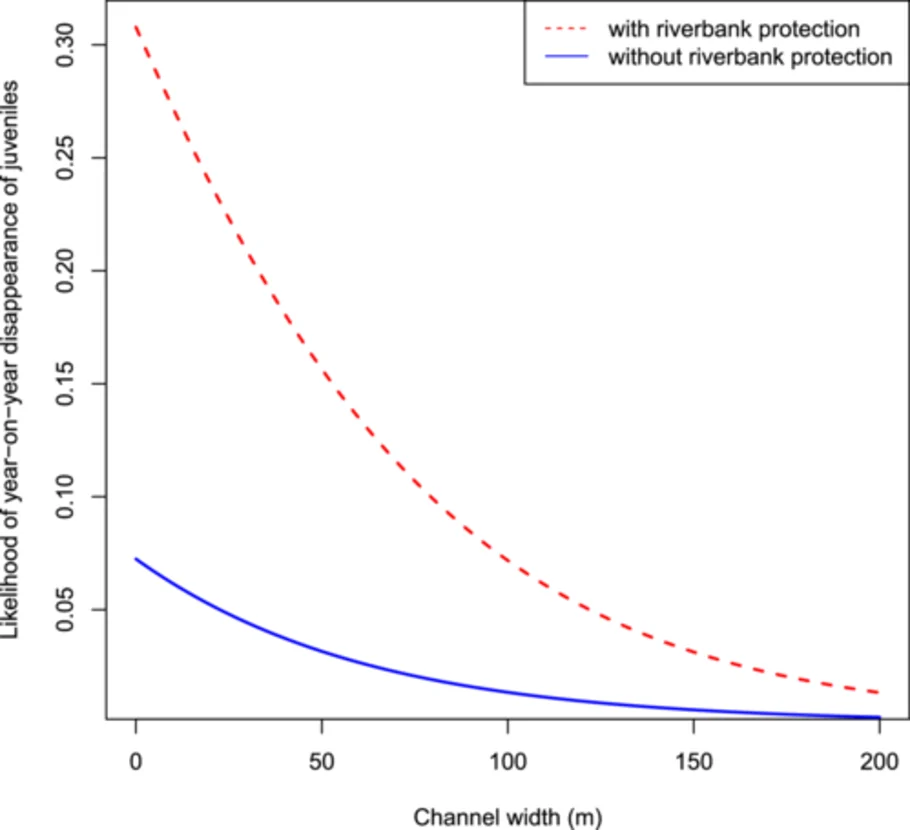
Graph depicting the probability of year‐on‐year disappearance of juveniles, in rivers with and without riverbank protection depending on the width of the river channel, according to the model of Equation 3.

For population survival, we obtained the logistic regression model with the optimum AIC value:

(3) $$\text{Prob}\left(\text{disappearance}\right)≔\text{sigmoid}(-0.0175\;\cdot \;\text{Width}+1.74\;\cdot \;1_{RBP}-2.55)$$

under the assumption that the deviance difference follows a *χ* ^2^‐distribution with 1 degree of freedom (as per Wilks’ theorem), analysis of deviances yielded $\chi_{1}^{2}=4.31,P=0.0379$ for the inclusion of riverbank protection and$\;\chi_{1}^{2}=4.43,P=0.0354$ for the inclusion of channel width. The resulting model is shown in Figure 5.

### Channel width

For channel width, we obtained the logistic regression model with the optimum AIC value, in which the natural logarithm of the channel width (in meters) is normally distributed with mean:

(4) $$0.484\cdot 1_{\text{Protected}}-1.85\cdot 1_{\text{CRB}}-0.0054\cdot \text{Altitude}+1.774\times 10^{-6}\cdot \text{Altitud}e^{2}+0.00183\cdot (\text{Altitude }\cdot 1_{\text{CRB}})+6.67$$

with estimated residual standard deviation 0.729 and *R* ^2^ = 0.735.

**Figure 6:**
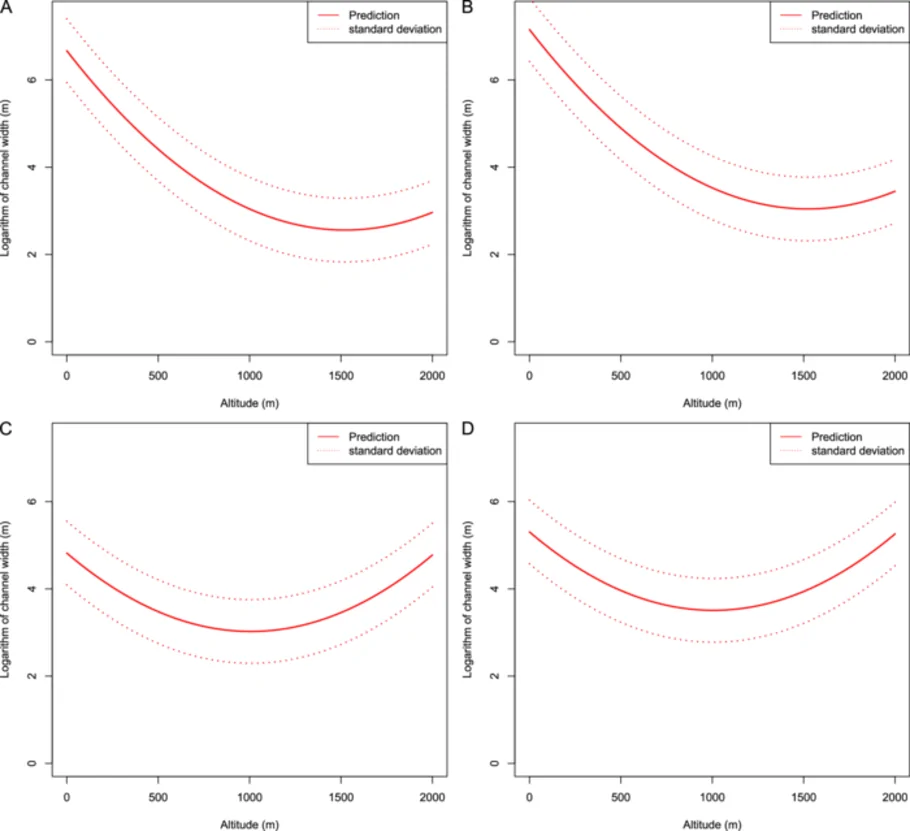
Graphs showing the relationship between channel width and altitude, according to the model of Equation 4. (A) Model for rivers with neither protected status nor concrete riverbanks. (B) Model for rivers with protected status and without concrete riverbanks. (C) Model for rivers without protected status and with concrete riverbanks. (D) Model for rivers with protected status and concrete riverbanks.

*F*‐tests were used to assess the inclusion of individual terms in the model. The *F*‐statistics and *P*‐values of these terms can be seen in Table 2, and the resulting model is shown in Figure 6. Appendix S1 illustrates the quadratic dependency on altitude using the simplified model with altitude as the only feature.

**Table 2:** Statistics obtained using *F*‐tests to assess the inclusion of individual terms in Equation 4.

Term(s)	*F*‐statistic	*P*‐value
CRB × altitude interaction	*F*_1,123_ = 13.02	*P* = 0.0004
Protected status	*F*_1,123_ = 10.67	*P* = 0.0014
CRB (main and interaction effects)	*F*_2,123_ = 7.66	*P* = 0.0012
Altitude (all terms)	*F*_3,123_ = 105.77	*P* < 0.0001
Altitude (squared term only)	*F*_1,123_ = 34.30	*P* < 0.0001

*Note*: CRB = concrete riverbank protection.

### Association between human factors

For the association between the presence of a check dam and protected status, Pearson's $\chi_{1}^{2}=7.72,P=0.0055$, and Cramér's V is 0.244. For the association between the presence of check dams and riverbank protections (viewed here as a variable with three levels: none, plain, and concrete), Pearson's $\chi_{2}^{2}=11.24,P=0.0036$, and Cramér's V is 0.294. For the association between protected status and riverbank protections, Pearson's $\chi_{2}^{2}=2.77,P=0.250$, and Cramér's V is 0.146. We also evaluated the correlation of the presence of check dams with the existence of any riverbank protection, obtaining Pearson's $\chi_{1}^{2}=1.60,P=0.206$, and Cramér's V to be 0.111, and with the presence of concrete riverbanks, obtaining Pearson's $\chi_{1}^{2}=12.39,P=0.0004$, and Cramér's V to be 0.309.

## DISCUSSION

### Constructing the final causal diagram

Along the Alpine rivers of northern Italy, the dynamics of juvenile *M. germanica* are linked to active channel width, which increases the availability of regularly and naturally disturbed gravel bars for germination. Narrower channels host fewer plants, with juveniles particularly sensitive to this constraint. Given that human alterations (concrete bank protections, dams, and channelization) tend to narrow channels and disrupt sediment or flow regimes, they reduce recruitment chance. Conversely, protected areas generally retain wider, more dynamic channels and better recruitment conditions. Altitude shapes channel size and hydrological regime (non‐linearly in our dataset) and also modulates phenology and water supply across the seasons, affecting the timing and success of germination and early growth. Where regulation has reduced extreme disturbance, distance from check dams can still influence juvenile presence by mediating local sediment pulses (Sitzia et al., 2016).

In this study, we used the limited data available to assess the factors determining the year‐by‐year likelihood of disappearance of juveniles in *M. germanica* populations by fitting a logistic regression model. Initially, we included the riverbank, check dams, protected status, altitude, and width of the active channel in this model. After checking all the different combinations of feature variables, our results demonstrate that the channel width and presence of reinforced riverbanks are significant factors, supported by both the AIC and a likelihood‐ratio test with *P*‐values of 0.0354 and 0.0379, respectively. This would be consistent with a causal diagram without any arrows from altitude, check dams, and protected status to plant survival, as illustrated in Figure 4C. We emphasize that this does not mean that we have confirmed the absence of such effects, which would be tantamount to confirming the null hypothesis. However, the fact that our dataset is unable to pick up those effects is consistent with their absence and does suggest that if they are present, their magnitude may be small compared to the effects mediated by the width of the active channel. This also supports the findings of Sitzia et al. (2016), who found a strong effect of the width of the active channel on the number of adult and juvenile plants along the Avisio River, incorporating a subset of the data used here.

We also investigated the possible influences affecting the width of the active channel. As a first approximation, we fitted a log‐linear regression model that also includes those sites in our dataset that did not have any recorded populations of *M. germanica* during the study period or where plant counts were not conducted in consecutive years. Our results suggest that a key factor for determining the channel width is (unsurprisingly) the altitude of the study site. In our dataset, the relationship is (log‐)quadratic rather than linear (Appendix S1).

Some of the spread in this graph can be explained by the anthropogenic factors included in the regression analysis, with the remaining residual variance of 0.739 reflecting natural variation in river sizes even at similar altitudes. Our data seem to suggest that concrete riverbanks are associated with narrower active channels, particularly at lower altitudes, while protected areas generally have wider channels than non‐protected areas. To better judge the reliability of those conclusions, we investigated the correlations between the variables involved in human activity. Pearson's *χ* ^2^ test found evidence of correlation between the presence of check dams and both the protected status of a site and riverbank protection. However, there is no evidence of correlation between the protected status and the protection of riverbanks. Cramér's V, which is around 0.3 for the visible correlations, suggests that while the association is clearly detectable, it is not deterministic or nearly so. Thus, there is still sufficient variation in both variables for their effects to be clearly distinguishable under linear regression.

In the causal framework captured in Figure 4C, the log‐linear regression model estimates the direct effect of altitude and human factors on the width of the active channel. However, there are two key limitations to this conclusion. First, while the variables included in this study reflect clear human impacts, other activities (e.g., sediment mining) have also shaped the river systems where *M. germanica* occurs (Surian et al., 2009; Ziliani and Surian, 2012). These pressures are often interrelated, driven by settlement and agriculture (Figure 4C, “human factors”), and may confound the effects attributed to the specific structures considered here. This is supported by Sitzia et al. (2023), who showed that the greatest influence of geomorphic properties on vegetation occurs at intermediate levels of human disturbance, suggesting that broader, unmeasured human influences may also play a role. Second, the causal diagram in Figure 4A assumes that human activity has not itself been affected by river morphology. However, human settlement patterns have long been determined by the rivers around them, and furthermore the demands of engineering could suggest preferential construction in narrower stretches of river. This can be addressed by refining the conceptual model of Figure 4A, leading to the conceptual model of Figure 4D.

### Implications of the model: An invitation for collaboration

Our model serves as an example of how causal diagrams can provide a useful framework for tackling the complex, interdisciplinary problems typical of conservation biology. The causal diagram provides a clear division between the different areas of science that are relevant to our study. On the one hand, plant scientists study the impact of various factors on the survival of feasible *M. germanica* populations. On the other hand, geomorphologists assess the impact of various extrinsic factors on the width of the active channel; they assess the incoming arrows into the node “channel width” in the original model of Figure 4A, and into the nodes “channel width” and “pre‐industrial channel width” in the refined model of Figure 4D. In terms of our diagrams, a geomorphologist would assess the incoming arrows on the node “*M. germanica* survival” (Figure 4B–D). Taken together, these efforts allow us to assess the effect of human actions on *M. germanica* survival. Completing the diagram would involve a third area of science, social sciences, to assess the role of geographic factors for human activity (the incoming arrows of the node “human activity”). A key advantage of the clear division into sub‐problems achieved by the use of causal diagrams is that we can use data from other river systems to evaluate the factors determining channel width. This includes systems for which either there are no usable data on *M. germanica* populations or there are no populations in the first place. As *M. germanica* is only present on a minority of northern Italian Alpine river stretches, this vastly increases the possible data sources.

### Causal diagrams as a language of ecology

In this case study, we use *M. germanica* habitats in Italy to illustrate how causal diagrams can be used to investigate survival or abundance of organisms, but we emphasize that the general principle of using causal diagrams can be applied broadly to conservation and applied ecology. The initial diagram of Figure 4A used for our case study could be adapted to elucidate survival or abundance of other alpine riparian species in similar habitat types, for example, other species in the genus *Myricaria*, or associated plants like *Hippophae* L. and *Salix* species, because the factors used here are likely to be important factors affecting these organisms. The variables selected, in this case channel width, presence of dams, and so on, are specific to riparian environments, but the general framework to draw causal diagrams that we describe here can be used to elucidate factors affecting the survival or abundance of any study organism in its habitat. For example, variables such as climatic data, habitat area or habitat fragment, presence of other species, or different human factors could be selected.

This contribution illustrates the potential of Pearl's causality as a tool for understanding and analyzing ecological data in different contexts. We believe that a key advantage of causal diagrams involves the modularity of the model; this allows complex systems to be subdivided into flexible components. Causal diagrams also make the assumptions of both the model and the confounding variables very transparent. Thus, causal diagrams can be understood relatively intuitively, allowing the qualitative assumptions, hypotheses, and outcomes of research to be assessed more easily by others. The use of causal diagrams can also be one element in addressing the current reproducibility crisis in ecology (Oza, 2023; Gould et al., 2025).

MacLeod and Nagatsu (2018) make the interesting case that the standard practices in ecology differ from those of some other scientific disciplines. In ecology, the thrust of scientific progress comes from datasets and statistical testing (data‐driven research). In contrast, fields like economics and physics approach research questions by developing a theoretical framework and then devising the experiments to test those hypotheses (theory‐driven research). This contrast in scientific philosophy is presented as a significant factor in making interdisciplinary research between ecology and other fields more difficult. Interdisciplinary research can be seen as lacking in structure (Beichler et al., 2014; Pujadas Botey et al., 2014); therefore, causal language can be instrumental in bridging fields, allowing for greater interdisciplinary research for conservation and environmental policy.

### Future directions of research

The present contribution paves the way for a more in‐depth study of the factors influencing channel width—a key factor affecting the survival of *M. germanica* populations. Such work can follow the refined causal diagram of Figure 4D and should adopt a more holistic view of human action than our dataset allowed. The large body of work investigating such issues suggests that such a deeper, causal understanding of the factors involved in changing channel morphology is feasible (Rinaldi et al., 2005; Surian et al., 2009; Ziliani and Surian, 2012, 2016; Huber and Huggenberger, 2015). Such an interdisciplinary partnership will result in a theoretically grounded model for the survival of *M. germanica* populations in northern Italy under clear, qualitative assumptions and backed by a larger body of data than ecological studies alone could provide.

However, understanding the complex mechanisms driving biodiversity loss and ecosystem function is just the first step towards action. By representing effects rather than mere associations, the assumptions behind a causal diagram ensure that a regression model is still valid after altering its features directly. Therefore, given a causal diagram and support for the relevant models, one can predict the effect of changing a feature, such as re‐naturing or protecting an area, by assessing that feature's effect on channel morphology and, in turn, the effect of that change on population survival. Thus, causal diagrams aid the transition from foundational research to policy and decision support. Pearl (2000) even describes a ladder of causality that reaches beyond interventions to counterfactuals, enabling scientists to think about situations in the conditional tense—what *would have* happened had we intervened. This could be useful for assessing policy decisions after the fact.

In the meantime, causal diagrams can be used to analyze larger ecological datasets with a variety of data types. Examples include the outcome of the Bottoms‐Up project, an EU‐funded forestry database built through international collaboration (Burrascano et al., 2023). We hope that the introduction of Pearl's causality and causal diagrams can facilitate collaborative and interdisciplinary research in applied ecology.

## AUTHOR CONTRIBUTIONS

K.W., K.R., and F.W. conceptualized the research; T.S. and B.M. collected the data; K.W. and S.W. acquired the funding contributing to the causality analysis; K.W. coordinated the project team; S.W. and F.W. provided guidance and supervision; F.W. analyzed the data; K.W., K.R., and F.W. drafted the manuscript. T.S. and S.W. reviewed and improved the draft manuscript, and all authors approved the final version.

## Supporting information


**Appendix S1.** Graph depicting linear and non‐linear models of the relationship between logarithmic channel width and altitude, showing that a quadratic relationship fits the data better than a linear one.

## Data Availability

All the code used in this study is available on Zenodo and GitHub (doi.org/10.5281/zenodo.16747701; Weitkämper, 2025). All the data used in this study is available on Research Data UNIPD (https://researchdata.cab.unipd.it/1622/; Michielon and Sitzia, 2025).
