# Supplementary material for: Pearl's causality for integrating ecological datasets: A case study on Myricaria germanica in northern Italy
Source: Appl Plant Sci. 2026 May 10;14(4):e70056. doi: 10.1002/aps3.70056 (PMC13495978; doi:10.1002/aps3.70056)

DOI 10.1002/aps3.70056

APPENDIX S1. Graph depicting linear and non-linear models of the relationship between logarithmic channel width and altitude, showing that a quadratic relationship fits the data better than a linear one.

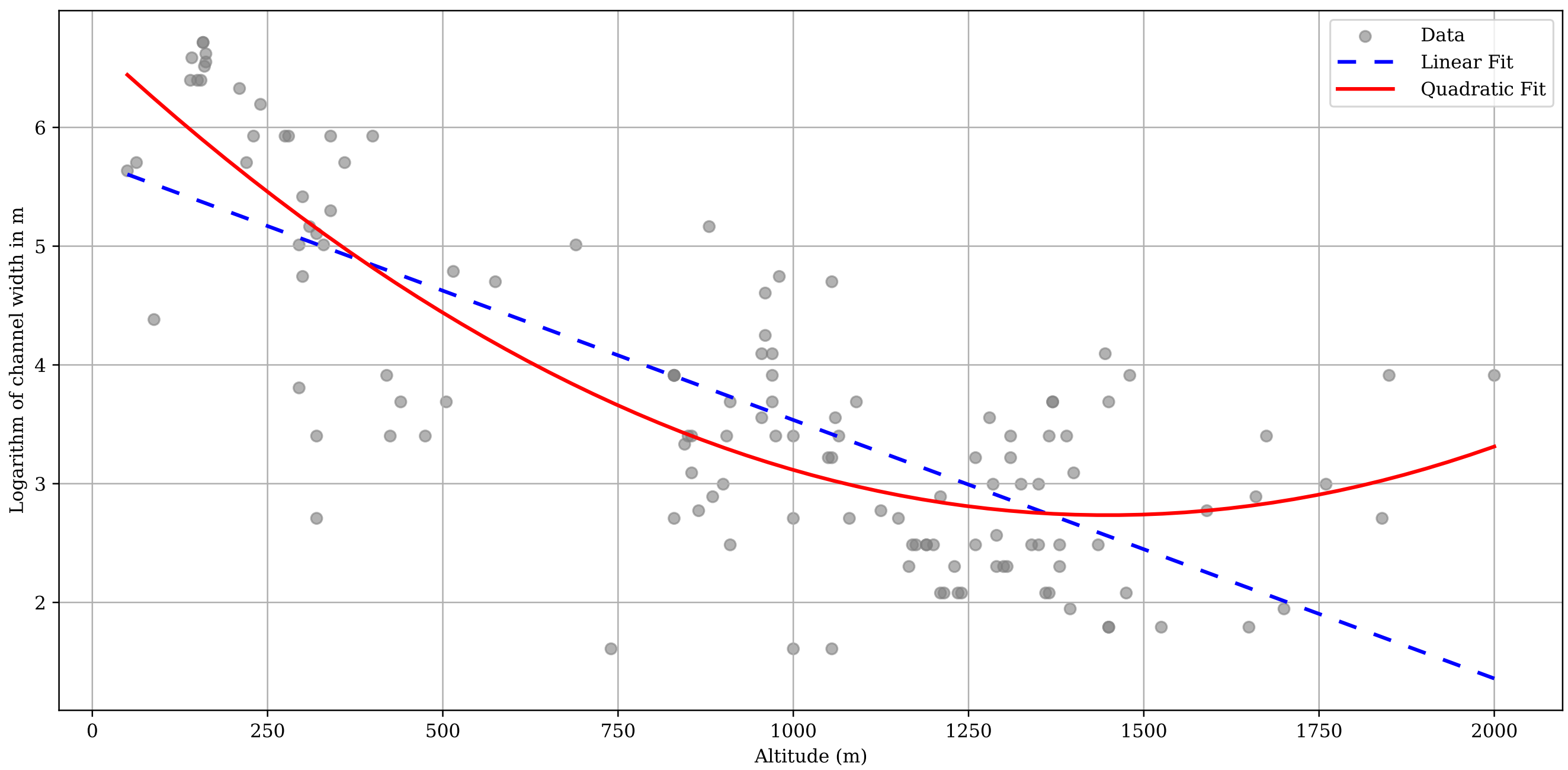

Supplement: Supplementary file 1 [file APS3-14-e70056-s001.pdf]
